# Challenges to Bringing Personalized Medicine to a Low-Resource Setting in Peru

**DOI:** 10.3390/ijerph18041470

**Published:** 2021-02-04

**Authors:** Nelly Solis, Elizabeth Zavaleta, Patrik Wernhoff, Constantino Dominguez-Barrera, Mev Dominguez-Valentin

**Affiliations:** 1 Universidad Católica Los Ángeles de Chimbote, Instituto de Investigación, Chimbote 02804, Peru; asolisv@uladech.edu.pe (N.S.); ezavaletal@uladech.edu.pe (E.Z.); 2 Department of Medical Genetics, Institute of Clinical Medicine, University of Oslo and Oslo University Hospital, 0424 Oslo, Norway; patrikw@me.com; 3 Independent Researcher, Lima Lima 39, Peru; c_dominguezb@yahoo.es; 4 Department of Tumor Biology, Oslo University Hospital, The Norwegian Radium Hospital, 0379 Oslo, Norway

**Keywords:** precision medicine, low-resource setting, vulnerable population

## Abstract

We provide an overview of the challenges that low-resource setting cities are facing, including a lack of global implementation of cancer screening programs, accurate data and statistics that may aid the health authorities and guide future public health activities, as well as reorient strategies, interventions and budgets to promote lifestyles that help prevent disease. Current cancer care does not fully reflect ethnic, cultural, environmental and resource differences. Herein, we described a snapshot of the cancer mortality and morbidity from a hospital that cares a rural and low-income population from Peru, called Chimbote (316,966 inhabitants) and showed the limitation of access to oncological care and genetic services. The city is located in the region of Ancash, which is a department of Northern Peru. Of note, we identified a greater proportion of cancer cases than previously described, with a young age of onset and differential profile of the most frequent cancers. With the emergence of increasingly effective interventions, it becomes paramount that populations living in resource-limited settings have access to cancer services and participate in genetics and genomic research.

## 1. Background

Cancer registries represent a resource for researchers to analyse the cancer burden, assess differences between cancer treatments and allow national cancer control planning in the context of the country’s sociocultural environment and health-care system. However, in Latin America and the Caribbean, there is a low level of reporting of cancer cases, and insufficient organization and funding of cancer registries compared to Europe and the United States. Only 6% of the Latin American and Caribbean population is covered by population-based cancer registries, compared with 96% of the United States and 32% of European populations [1,2].

Peru, like several other Latin American countries, is experiencing remarkable population growth. The current population is 33,010,043 as of 2 August 2020, based on a Worldometer elaboration of the latest United Nations data [3]. There are only two cities with population-based cancer registries (Lima and Arequipa). The global burden of cancer in Peru is rising, with 66,000 new cases (and 130 deaths per 100,000 inhabitants) in 2018 [4]. According to Globocan 2018 [5], prostate and breast cancer are the most frequent cancers in men and women, respectively. However, infection-associated cancers (cervix and stomach) are also common and rank highest in the national cancer mortality profile. The high rates of cancer incidence and cancer deaths could be associated with lifestyle behaviours, including diet, physical inactivity and obesity, but may also reflect the limited availability of screening programmes, early diagnosis, and curative treatment programmes. This is the result of the suboptimal organisation of national health systems, as well as social, cultural, and economic inequalities in the country [1,6,7,8].

At the Latin America level, the majority of the countries lack adequate systematic screening or prevention programmes. It is widely accepted that general screening of people above 50 years is a cost-effective and efficacious way of reducing colorectal cancer (CRC) [9,10]. 

Several Central and South American countries have developed national guidelines for early detection (management and follow-up) of CRC; however, screening programs in the region are uncommon [9,11]. Of them, Argentina and Uruguay have implemented CRC screening programs while Brazil, Chile, Cuba and Ecuador have initiated pilot studies. Some initiatives have emerged in some states of Mexico and awareness campaigns have been carried out in Bolivia, Colombia, Costa Rica, Peru and Venezuela [12].

## 2. Snapshot of the Current Cancer Care in Low-Resource Setting Cities

Herein, we would like to add some data from Latin America in regard to the currently incomplete picture of the risk attributable to inherited, environmental, or lifestyle factors for cancer in most of the regions and rural areas from Peru. Herein, we provide, for the first time, a snapshot of the morbidity, mortality and cancer pattern from a regional hospital that cares for the rural and low-income population from Northern Peru (Chimbote), where morbidity, mortality and cancer profile are not in line with the national reports [13]. Of note, cancer screening methods are not fully implemented, and genetic services are scarce. The city is located in the region of Ancash, that is a department of Northern Peru. 

La Caleta Hospital (category II) is one of the three main hospitals in Chimbote and has 100 beds. The hospital provides comprehensive health services (preventive, promotional, recuperative and rehabilitative), but does not have an oncology service, specialized medical personnel or equipment to treat cancer patients. We assessed morbidity and mortality records based on organ system rather than by specific diseases. During the last five years (2014–2019), a total of 464,473 patients were registered. Poorly defined signs/symptoms and stages represented 16% of all morbidities, followed by the diseases of the respiratory system (12%), diseases of the nervous system and senses (9.9%) and injuries and poisonings (8.4%). The intra-hospital mortality rate was 3.8 per 1000 users. Out of a total of 728 deaths, the highest cause of mortality was diseases of the respiratory system (24%), followed by the circulatory system diseases (17%), infections (14%) and digestive system diseases (10%). Interestingly, tumours were recorded with a low frequency (2%). In total, 381 patients were diagnosed with a neoplasm, where 76% (289/381) were benign, 12% malignant (12%, 47/389), 4% (4%, 14/381) aplasias and other anaemias and (3%, 12/381) neoplasms in situ. When analysing the malignant neoplasms (*n* = 47), the most frequent neoplasms were reported for the submaxillary gland (7/47, 14.9%), stomach cancer (6/47, 12.8%), endometrial cancer (5/47, 10.6%), lymphocytic leukaemia and brain tumours (3/47, 6.4% each), followed by liver and breast cancer (2/47, 4.3% each), myeloid leukaemia (2/47, 4.3%) and unspecified leukaemia (2/47, 4.3%). Cancer stage information was available for 6 (13%) of the patients who had stage III cancer. Females were more often reported to have cancer than males and the age at diagnosis was under 55 years in 32/47 (68%) of the cases. Often, early age of onset is helpful for identifying patients who are affected by inherited syndromes and carry a pathogenic germline variant associated with cancer predisposition. It is also known that most hereditary cancer mutations confer susceptibility to cancers in multiple organs [14]. The identification of individuals harbouring variants in cancer-associated genes may allow more appropriate risk management, a better knowledge of associated manifestations and a more accurate clinical and family history management. The local implementation of genetic and genomic research will provide insight into the aetiology of cancers and improve knowledge of cancer susceptibility through the identification of germline pathogenic variants, perhaps including novel variants unique to the Peruvian population. The Peruvian population is a multi-ethnic population with Amerindian (45%), Mestizo (37%), and white Spanish influence (15%), along with the presence of other minority ethnic groups, such as African American, Japanese, and Chinese (3%) [15]. Therefore, a recent study [16] reported that Mestizo populations have 60%–70% native genes, but in some geographical locations, it may reach up to 80%. The study reported a high number of new SNPs from Peruvian population, demonstrating a high Native ancestry component, even as compared to the results obtain from the Mexican genomic project [16]. Local, national and international scientific collaborative studies will enhance the genetic research in Peru, despite the lack of national investment. Importantly, the access to the healthcare providers, government and policymakers is relevant for its translation to the public health system, that will aid the implementation of precision medicine [17]. 

Of note, according to the Pan American Health Organization, neoplasms represented 20% of the proportional mortalities (% of total deaths, all ages, both sexes) in 2014 [18]. However, we described a 10-times lower frequency of tumours as a cause of mortality. Screening programs can reduce the cancer mortality rate by at least 25% (e.g., breast cancer screening by mammography that has been the norm in many countries for the last four decades), however there are still hospitals in Peru (like La Caleta), where these screening methods are not fully implemented. A low percentage (7.4%) of cancer cases were detected by the current programs for the early detection of cancer in Peru [19]. Correspondingly, 75% of cancer patients were first diagnosed at an advanced stage of the disease [19]. Late-stage cancers often lack an effective treatment option, and the survival rates increase significantly when cancer is identified at early stages, as the tumour can be surgically removed or treated with less toxic drug regimens. It is therefore imperative to promote better preventive measures, improve overall health and lower costs for patients to ensure their access to holistic, yet personalized, medical care and social care services throughout their lives. According to the Northern Peru Cancer Registry (IREN Norte), 15,435 cases of neoplasms have been described in 2007–2018, the most frequent being: cervix (2038, 13.20%), breast (1934, 12.53%), stomach (1587, 10.28%), skin, no melanoma (1527, 9.89%) and prostate (1221, 7.91%) [4,20,21,22]. Importantly, we described, for the first time, the stomach and the submaxillary gland cancers as the most frequent cancers from a local hospital and 68% of the cases had an early age of onset. Importantly, further studies analysing large series of cancer patients from this geographic region will be necessary to accurately estimate the prevalence and the relevance of the early age of cancer onset in this population.

### Challenges and Initiatives 

Current cancer care does not fully reflect ethnic, cultural, environmental and resource differences from the Peruvian population. There is a lack of initiatives to ensure equitable and global access to cancer screening and treatment in rural and low-income populations from the country. For example, cancer patients from Chimbote need to travel to the larger cities, such as Lima (capital, 428 km/266 miles) or Trujillo (132 km/82 miles), in order to initiate their treatments. An ongoing study identified that the waiting time for cancer patients to get access to cancer treatment is from 1–3 years [21], which has an impact on the prognosis, treatment and quality of life of the patient. 

In addition, we noted inaccurate pathological diagnosis reports and a lack of electronic data in the local hospitals, which make the follow-up of these patients a challenge. We may assume that most of the cancer patients reported here are not included in the national cancer statistics from Peru. In Peru, the cancer registries and programmes for cancer prevention are still immature. More accurate data may aid the health authorities and guide future public health activities, as well as reorient strategies, interventions and budgets to promote lifestyles that help prevent disease. With the emergence of increasingly effective interventions, it becomes paramount that populations living in resource-limited settings can access cancer services.

Some of the barriers that low-setting cities from Peru are facing include the lack of cancer programs in rural regions, a limited number of adequately trained health care professionals, non-availability of genetic testing at the public health care system and the lack of insurance coverage for such genetic tests. Furthermore, the lack of supportive healthcare policies, limited awareness about genomic medicine, hereditary cancer and its risk by patients and physicians, few educational opportunities in cancer genetics, and the lack of infrastructure constitute some of the challenges for these cities. In general, in the Latin American countries, training in human genetics and medical genetics is scarce. We have previously reported that genetic counselling is mainly offered by medical geneticists and other specialists with training in genetics [23]. However, there are ongoing initiatives, including national and international collaborations that aim to ensure that all Latin Americans countries (including Peru) may have access to genetic services and to bring additional awareness to medical professionals and public health leaders [23].

In this situation, actions need to be planned and implemented in Peru, such as addressing intervention research for the quality of care related to cancer diagnosis, treatment and/or survivorship, improving early cancer detection in all geographical areas of Peru, in order to more effectively reduce cancer-related mortality, and to implement an information and communication technology (ICT) infrastructure for data management and the reporting of cancer patient data. This should allow improved access to early cancer care, with particular attention to rural and underserved populations. In the direction of implementing precision medicine in Peru, there is a need for the translation of research to the public health systems. This implementation is expected to dramatically improve patient outcomes, reduce the suffering of patients and their families from preventable cancers and reduce health care costs through a shift in focus from disease treatment to prevention and individualized therapies. 

## 3. Conclusions

We described the cancer mortality and morbidity from a local hospital in Northern Peru. We identified a greater proportion of cases than previously described, with a young age of onset. Current cancer care does not fully reflect ethnic, cultural, environmental and resource differences. Our aim was to describe the cancer situation in a low-resource setting and better describe the needs of this population, however larger series are necessary to validate our findings. In Peru, the cancer registries and programmes for cancer prevention are still immature. More accurate data may aid the health authorities and guide future public health activities, as well as reorient strategies, interventions and budgets to promote lifestyles that help prevent disease. With the emergence of increasingly effective interventions, it becomes paramount that populations living in resource-limited settings can access cancer services.
